# Electrically Tunable Geometric-Phase Optical Element with Hybrid Retardation and Subwavelength Pixel Size Enabled by Metasurface-Induced Liquid Crystal Patterning

**DOI:** 10.1021/acsaom.5c00177

**Published:** 2025-07-16

**Authors:** Xin Chang, Mike Pivnenko, Weijie Wu, Yayan Tan, Pawan Shrestha, Daping Chu

**Affiliations:** † Centre for Photonic Devices and Sensors, 2152 University of Cambridge, 9 JJ Thomson Avenue, Cambridge CB3 0FA, United Kingdom; ‡ Cambridge University Nanjing Centre of Technology and Innovation, Site A, 23 Rongyue Road, Jiangbei New Area, Nanjing 210000, China; § AllFocal Optics, St John’s Innovation Centre, Cowley Road, Cambridge CB4 0WS, United Kingdom

**Keywords:** active metasurface, geometric phase, hybrid retardation, liquid crystal alignment, fast switching

## Abstract

In this work, we present an electrically tunable hybrid geometric phase optical element (GPOE) at a telecommunication wavelength with the geometric phase jointly imparted by a liquid crystal (LC) layer and the embedded metasurface. A geometric phase (GP) grating was demonstrated both numerically and experimentally, achieving a voltage-switchable diffraction efficiency ranging from 8% to 56% at 1550 nm. Notably, LC patterning was induced exclusively by the spatially varying metasurface with a metaatom period of 800 nm, enabling the realization of LC-GPOEs with a subwavelength pixel size. Furthermore, the metasurface can be purposely designed to provide dynamic light modulation by leveraging LC-mediated resonance tuning of the metasurface, which paves the way for advanced optoelectronic devices. In this work, a halfwave condition was realized through the propagation phase and the resonance phase, which were provided by the LC and metasurface, respectively. As a result, the LC-GPOEs can be made very thin compared with conventional LC-GPOEs. Eventually, the proposed device was able to work with a switching frequency exceeding 110 Hz.

## Graphical abstract

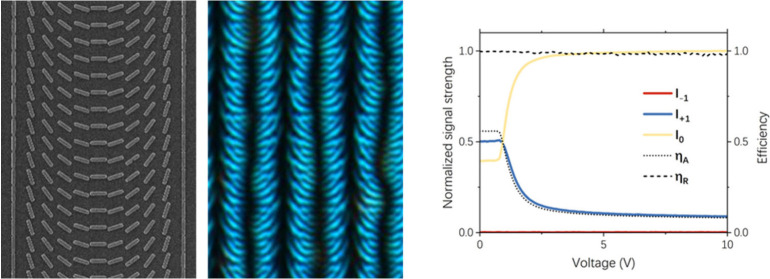

## Introduction

Geometric phase optical elements (GPOEs) are gaining traction in a variety of applications such as optical combiners for augmented reality (AR) glasses, beam-splitting gratings for differential interference contrast (DIC) microscopy, vector beam generators for laser processing, etc.^1–6^ Due to the spin–orbit interaction (SOI), GPOEs typically exhibit high efficiency and broadband response. Typically, GPOEs rely on optical anisotropy, which can be realized through multiple approaches. For instance, femtosecond laser pulse-induced nanogratings in glass have been used to create geometric phase (GP) gratings with high optical efficiency, high transmission, and high damage threshold.^3,4^ In addition, metasurface-based GPOEs, such as achromatic metalenses^7^ and polarization-dependent gratings,^8^ have emerged recently due to their high resolution and tailorable anisotropy. Moreover, GPOEs using liquid crystals (LCs) have been demonstrated in computer-generated holography (CGH)^9,10^ and diffractive waveguide combiners in AR glasses.^5^

For LC-based GPOEs (LC-GPOEs), the GP is provided by the spatially varying distribution of LC directors. This has been realized in several studies. For example, nanoscale LC alignment was realized by mechanically rubbing a polyimide using an atomic force microscope (AFM) operated in contact mode.^11^ LC alignment was created on the surface along the scanning direction. Besides, two-photon polymerization (TPP) was used to generate planar and volumetric structures with LC anchoring energy.^12^ Similarly, TPP was also used to generate stamps for nanoimprint lithography, which was further employed to replicate LC-aligning structures.^13^ Photopatterning is another mainstream approach that includes a broad category of various techniques. It has been widely adopted because it is free from physical contact, contamination, and electrostatic charge. In practice, photoalignment is implemented through interferometry and direct writing.^14^ The interferometry method employs circularly polarized reference and object beams to create the spatial distribution of the azimuth angle of the linearly polarized interference beam. For example, ref 15 reported a cascaded achromatic lens using cholesteric LC material, which was fabricated through photoalignment polarization holography (PAPH). The direct writing method, however, utilizes a motorized stage and an active polarization control module to write the prescribed orientation information. Similarly, a lithography-based approach was reported using digital mirror device (DMD),^16^ liquid crystal on silicon (LCoS),^9,17^ and a LC display (LCD) projector.^18^ A polarization controller is required in DMD and the LCD projector-based method. The LCoS-based approach provides a more elegant polarization modulation by transforming the phase profile loaded in the LCoS device into the polarization orientation of the output beam, using a 45° rotated quarter-wave plate. Other photoalignment methods include plasmonic metamasks utilizing nanoaperture-induced local polarization effect^19^ and scanning wave photopolymerization resulting from diffusion-induced mass flow.^20^

Recently, LC-based dynamic metasurfaces are gaining momentum.^15,21–25^ However, the precise alignment of LC around the metasurface remains a critical issue. Some works^22,26^ reported single-sided LC alignment, where conventional alignment methods (such as mechanical rubbing) were applied to the opposing substrate while the metasurface substrate was left untreated. Fine control over the orientation of the LC around metasurface has been achieved through photoalignment techniques^27–29^ and the application of an external magnetic field.^30^ Additionally, LC alignment induced directly by the metasurface itself has also been reported.^31–34^ In this work, metasurface-induced LC alignment was employed, and a hybrid GP grating device was demonstrated. The electrically tunable diffraction efficiency ranging from 8% to 56% at telecommunication wavelengths, along with a high operating frequency (>110 Hz), was validated. Compared to LC-GPOEs using conventional methods, the proposed GP device in this work features subwavelength pixel size (GP device pixel size of 800 nm) determined by the metasurface. As the metasurface can be precisely defined through the lithographic processes, smaller pixel size and spatially arbitrary LC alignment is feasible. The self-assembly of the LC around the metasurface provides a unique solution to complex LC patterning for LC-GPOEs, driving advancements in high-resolution reconfigurable optoelectronic devices.^9,35–39^ More importantly, halfwave retardation was realized in a hybrid manner through the propagation phase and resonance phase, provided by the LC and metasurface, respectively. This is beneficial for the reduced cell thickness compared to conventional LC-GPOEs where the half-wave condition was implemented through propagation-induced phase accumulation.

## Simulation and Design

The metasurface unit cell is composed of rectangular pillars with the following dimensions: width (*w*) = 160 nm, length (*l*) = 750 nm, metaatom period (*p*_*x*_ = *p*_*y*_ = *p*) = 800 nm, and height (*h*) = 190 nm, as shown in the inset (1) of Figure 1a. The dimensions were selected so that the phase retardation (δ = |φ_*x*_ – φ_*y*_|; φ_*x*_ and φ_*y*_ represent the phase delays of the *x*- and *y*-linearly polarized incident light after passing through the metasurface) is close to π at the wavelength of 1550 nm, and the average transmission (*T*_ave_) is high. The simulation was carried out with the commercial software COMSOL. The indices of silicon, the glass substrate, and the LC used in the simulation are *n*(Si) = 3.48, *n*(glass) = 1.50, *n*_o_(LC) = 1.51, and *n*_e_(LC) = 1.81, respectively. Due to the anchoring force provided by the metasurface, the LC is aligned along the long side of the metaatom. The simulation assumed homogeneous LC distribution around metaatoms within a single metaatom cell. Numerical simulations were performed for a unit cell with a cell gap of 1.5 μm, as is depicted in inset (1) of Figure 1a. The results for φ_*x*_, φ_*y*_, δ, and *T*_ave_ are plotted against the LC director angle (θ) in Figure 1b. The angle θ is defined with respect to the positive Y axis, as illustrated in Figure 1a. When the voltage is off (θ = 0°), the halfwave condition is met. As θ increases, the retardation decreases (blue solid line). It is also evident that φ_*y*_ (blue dashed line) is the main cause for LC-mediated retardation while φ_*x*_ (blue dotted line) is less affected. To further support this, the electric energy distribution when θ = 0° is shown in Figure 1c. A strong electric field is induced in the case of *E*_*y*_^in^, while the field enhancement for the case of *E*_*x*_^in^ is trivial. This implies that both the metasurface and the LC contribute to the overall retardation.

**Figure 1:**
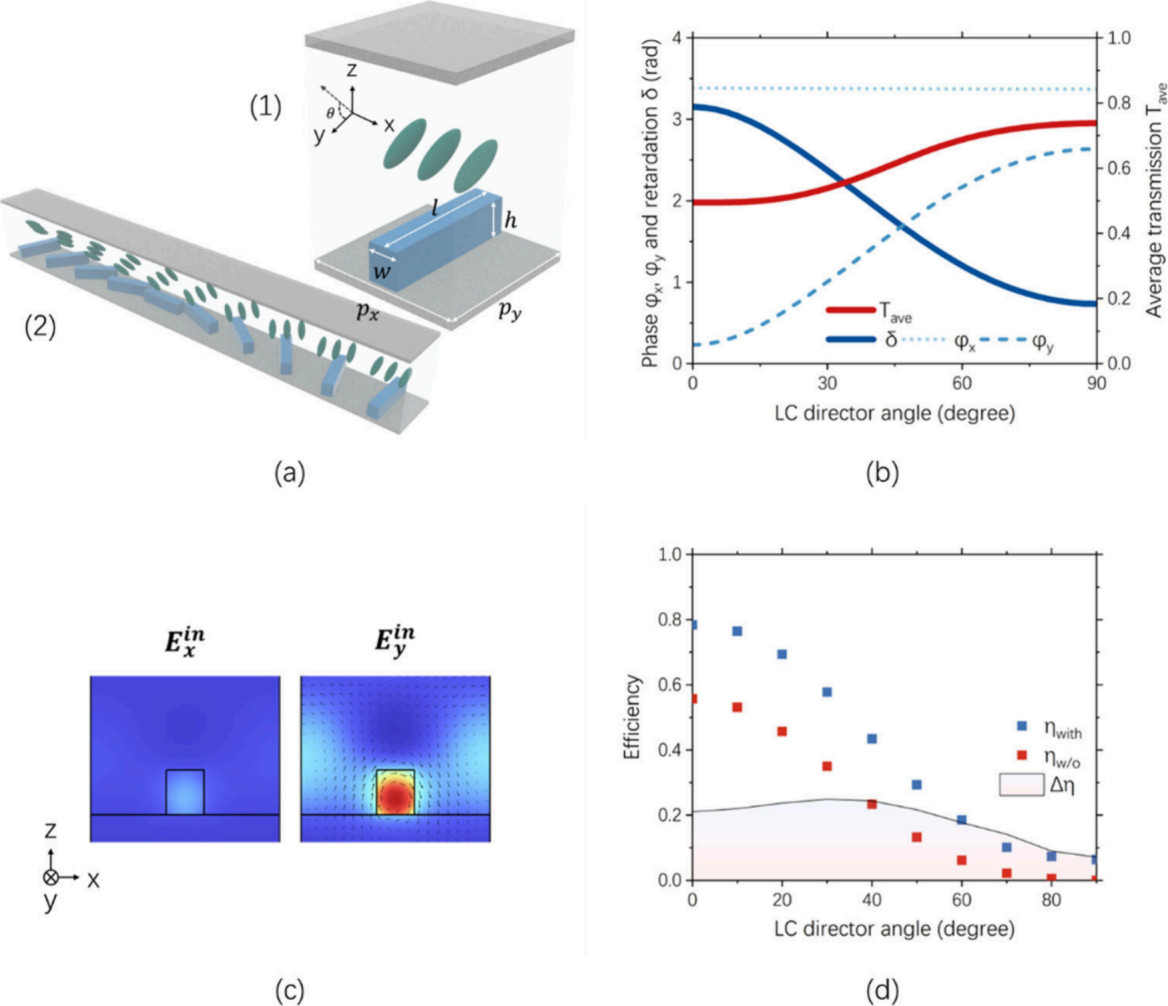
Simulation results. (a) Illustration of (1) unit cell and (2) GP grating. (b) Simulated retardation (δ), average transmission (*T*_ave_), phase delays (φ_*x*_ and φ_*y*_) against different LC director angles. (c) Electric energy distribution when the incident light is polarized along *x* and *y* directions. (d) Simulated diffraction efficiency against LC director angles with (η_with_) and without (η_w/o_) the metasurface.

To verify the joint contribution of the GP by the metasurface and the LC, a three-dimensional GP grating with 11 GP levels was numerically simulated. The GP grating is illustrated in inset (2) of Figure 1a. The diffraction efficiency of the GP grating (η_with_), defined as the fraction of the first order within all transmitted light, was calculated for different θ, as shown in Figure 1d. The maximum and minimum η_with_, approximately 80% and 6%, were obtained when the voltage was off (θ = 0°) and on (θ = 90°), respectively. As a comparison, the same simulation was performed with the metasurface removed, while keeping the LC directors unchanged. The efficiency η_w/o_ shows considerable reduction, as indicated by Δη = η_with_ – η_w/o_ in Figure 1d. The maximum Δη is >20%, confirming the contribution to the GP from both the metasurface and the LC. The reduction in efficiency after removing the metasurface is because the halfwave condition is no longer met by the LC alone. The use of the metasurface can therefore effectively reduce the device thickness while meeting the halfwave condition. As a result, the response time can be reduced, which is particularly valuable for long-wavelength applications. Furthermore, this comparison validated the hybrid nature of the phase retardation, which is jointly provided by the light propagation through the LC and resonance with the metasurface. The rotation of the LC director after applying the electrical voltage will simultaneously affect the retardation induced by propagation and resonance.

## Methods

The proposed GP grating was fabricated as follows. Amorphous silicon was deposited on an ITO-coated glass substrate via sputtering. Then the pattern was transferred to silicon via standard electron beam lithography and reactive ion etching. The scanning electron microscope image of the metasurface substrate is shown in Figure 2a. The cell was then assembled by pressing an ITO-coated glass superstrate against the metasurface substrate via spacers (1.5 μm in diameter, microparticles). No alignment treatment was applied to the superstrate, ensuring that the LC alignment was entirely provided by the metasurface. Finally, the cell was filled with a nematic LC (GT7-29000, Merck).

**Figure 2:**
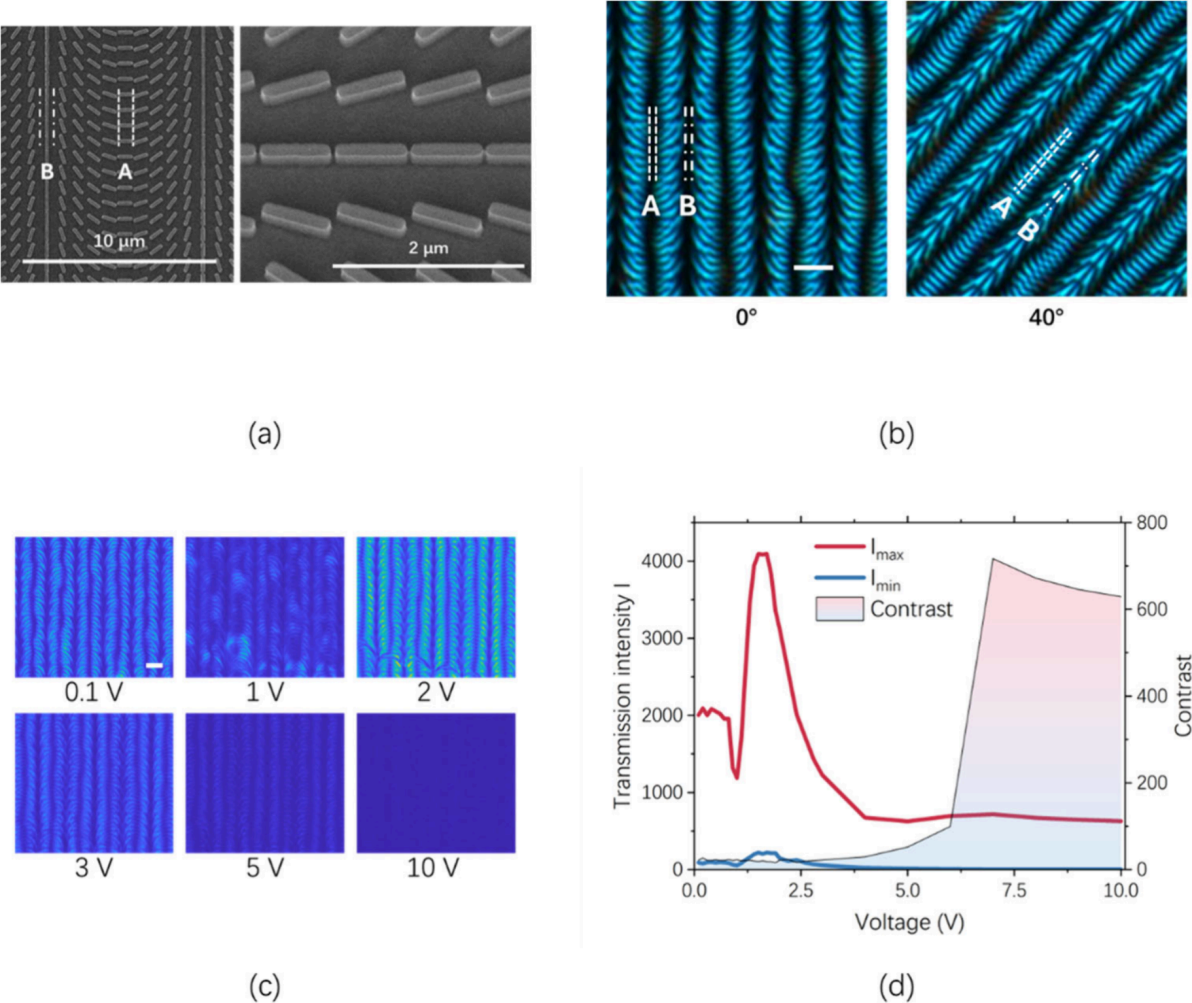
Characterization of LC alignment induced by the metasurface. (a) SEM image of the metasurface. (b) Microscopic image of the LC-filled cell under 0° and 40°. (c) Microscopic image of the LC-filled cell under various peak AC voltages. (d) Maximum and minimum transmission intensity (*I*_max_ and *I*_min_) and the contrast (*I*_max_/*I*_min_) under different voltages. The scale bars in panels b and c indicate a distance of 8 μm.

## Results and Discussion

The device was observed with a microscope using visible light between crossed polarizers when it was placed at α = 0° and α = 40°, as shown in Figure 2b. The measurement setup is identical to that in ref 27. A linear polarizer and cross-polarized analyzer were used before and after the metasurface cell. The metasurface cell is situated on a rotating stage so that the optical transmission through the metasurface cell can be observed at various in-plane angles. Optical transmission reaches a maximum when the LC directors are 45° with respect to both polarizer directions. Minimum transmission is obtained when the LC directors are parallel to either of the polarizer directions. When α = 0°, the image shows spatial variation in LC alignment direction, which is consistent with the orientation direction of metasurface pillars in Figure 2a. The dark strips (A and B) in Figure 2b correspond to the horizontal and vertical pillars in Figure 2a, indicating the good quality of the metasurface-induced LC alignment. In order to verify that the pattern observed in Figure 2b reflects the orientation of the LC, AC voltage was applied, and the variation of pattern was recorded, as shown in Figure 2c. When the peak voltage (V) was increased to 10 V, the pattern disappeared due to the vertical alignment of LC directors (θ = 90°). The maximum and minimum transmission intensity (*I*_max_ and *I*_min_) of the pattern, and the contrast (*I*_max_/*I*_min_) were extracted from each recorded image, as shown in Figure 2d. The profile of *I*_max_ exhibits a typical pattern for transmission through a LC-based waveplate, where the peak of *I*_max_ at *V* = 1.6 V represents the overall halfwave condition for the measuring wavelength. The maximum contrast is over 630, indicating the dominant role of the LC in this measurement.

Finally, the hybrid GP grating was characterized by a near-infrared laser, and the intensity of the diffracted light was measured with a beam profiler. Figure 3a shows the captured laser spots for linearly polarized (LP) and circularly polarized (CP) incident beams. The intensity of the +1st-order (*I*_+1_), −1st-order (*I*_–1_), and 0th-order (*I*_0_) diffracted beam were recorded for the CP 1550 nm incident beam at different voltages, as shown in Figure 3b. The +1st-order diffraction efficiencies η_A_ = *I*_+1_/(*I*_+1_ + *I*_0_ + *I*_–1_) and η_R_ = *I*_+1_/(*I*_+1_ + *I*_–1_) were also plotted in Figure 3b with dotted lines. When the applied voltage *V* = 0 V, η_A_ is approximately 56% while η_R_ exceeds 99.7%, indicating a high contrast. As *V* > 0.8 V, η_A_ drops rapidly and saturates around 8% due to the vertical alignment of LC directors. The highest η_A_ = 56% is lower than the numerically obtained value (∼80%) whereas the lowest η_A_ = 8% is close to the numerical value (∼6%). The discrepancy could arise from the distorted LC director distribution at the glass superstrate, where LC alignment was not prescribed. In addition, the diffraction efficiency was measured for several wavelengths from 1500 to 1630 nm, as shown in Figure 3c. There is a slight decrease in η_A_ (blue squares) as the wavelength increases. However, η_R_ (black squares) remains above 99% for all wavelengths when *V* = 0 V and above 95% when *V* = 10 V. As an electrically tunable device, the response time was measured for the +1st-order diffracted beam with the results shown in Figure 3d. The on and off times (τ_on_ and τ_off_) were measured to be 0.54 and 8.76 ms, ensuring the switching frequency of >110 Hz. The fast switching speed can be attributed to the hybrid retardation of the proposed device, which is provided by both the LC and the metasurface, as is indicated in Figure 1d. Consequently, the thickness of the LC can be made very thin and the response time can be effectively reduced. The cell gap in this case can be made even thinner, as the metasurface is only 190 nm in height.

**Figure 3:**
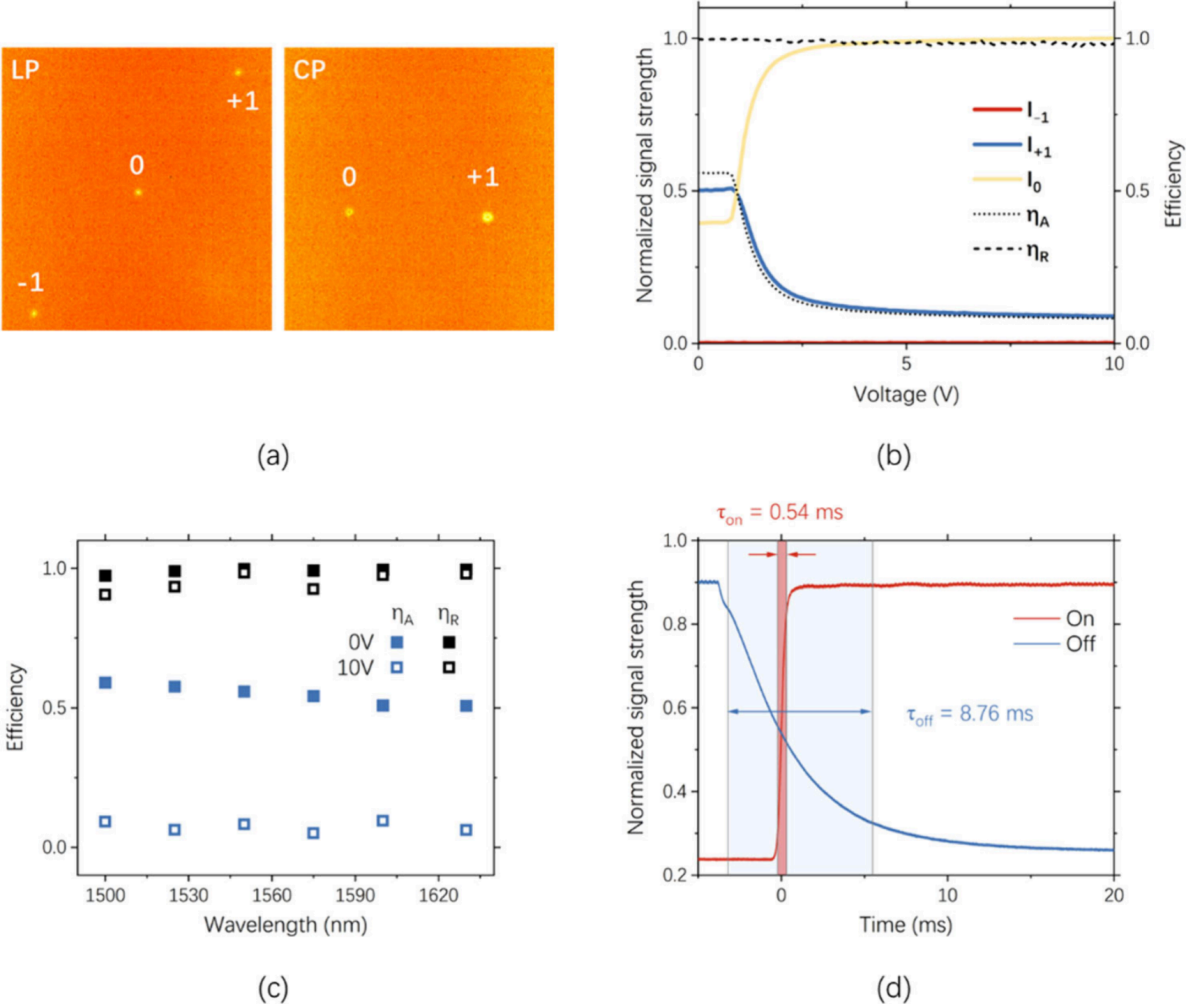
Performance characterization of electrically tunable hybrid GP grating. (a) Image of the diffracted beam spot. (b) Variation in the intensity (*I*_+1_, *I*_0_, *I*_–1_) and efficiency (η_A_ and η_R_) of the diffracted beam against the applied voltage. (c) Variation in diffraction efficiency (η_A_ and η_R_) with wavelengths. (d) Switching characteristics of the hybrid GP grating.

## Conclusion

In this work, an electrically tunable hybrid GP was demonstrated through a tunable GP grating. The LC alignment characteristics were measured, confirming the effective LC alignment induced solely by the metasurface. In addition, the electrical tunability showed efficiency modulation at 1550 nm with a tunable range between 8% and 56%. The modulation frequency was also measured to be >110 Hz. This work shows the benefits of aligning LCs with the metasurface nanostructures. A metasurface with a subwavelength metaatom period enables LC-GPOEs with subwavelength pixel size. It is expected that higher-resolution LC patterning with a smaller pixel size is possible by reducing the metaatom period below the wavelength in the visible spectrum. Meanwhile, the interaction between the metasurface and the LC provides a new degree of freedom for dynamic light modulation in LC-GPOEs. In this work, the hybrid retardation, provided by the light propagation within the LC and the electromagnetic resonance induced by the metasurface, was utilized to reduce the cell thickness and to increase the switching speed. Overall, this work demonstrates a novel route toward multifunctional LC-GPOEs with designer LC patterning.

## Data Availability

Data underlying the results presented in this paper are not publicly available at this time but may be obtained from the authors upon reasonable request.
